# Psychiatric rehabilitation

**DOI:** 10.4103/0019-5545.69250

**Published:** 2010-01

**Authors:** H. Chandrashekar, N. R. Prashanth, P. Kasthuri, S. Madhusudhan

**Affiliations:** Department of Psychiatry, Bangalore Medical College and Research Institute, Bangalore, India

**Keywords:** Rehabilitation, mentally ill, assessment

## Abstract

Psychiatric rehabilitation is an important component in the management of the mentally ill. This article presents a selective review of the publications in this journal. Questions addressed in this review range from assessment of rehabilitation needs to different rehabilitative approaches. Although the number of publications providing the answers is meager, there are innovative initiatives. There is a need for mental health professionals to publish the models they follow across the country.

## INTRODUCTION

Psychosocial rehabilitation is a therapeutic approach that encourages a mentally ill person to develop his or her fullest capacities through learning and environmental support.[1]

Psychiatric rehabilitation and psychiatric treatment are separate, yet equally important complementary components of mental healthcare. Even as psychiatric treatment (Pharmacological and psychological) aims at controlling psychiatric symptoms, psychiatric rehabilitation focuses on functioning and role outcomes. The new focus of rehabilitation is on wellness and optimum quality of life.

The rehabilitation program should start right from the first time the patient has come into contact with a mental health professional. A clinician waiting to start rehabilitation after the patient becomes asymptomatic, may not benefit the patient or the family in the long run.[2]

This article reviews publications in the IJP from its inception to date, in the area of rehabilitation. We have tried to summarize these articles and suggest future directions. This includes editorials, commentaries, review articles, book reviews, and case reports.

### Literature review

The first available publication regarding rehabilitation in our research is by Gupta *et al*.,[3] from the Hospital for Mental Diseases, Ranchi. They studied the possible causes and nature of chronicity among psychiatry patients and tired to categorize patients on the basis of residual assets (Functional ability). They found that 269 patients out of 450 (total bed strength) had stayed in the hospital for a period of longer than two years at a stretch. Thus 60% of the mental hospital beds were occupied by chronic patients. They evaluated their need of staying, and found that 6.5% of these chronic patients required full hospitalization, 71.75% required limited hospitalization and were capable of productive work under supervision, and 22% were there purely for social reasons and hospitalization was not necessary. They suggested rehabilitation services, including an occupational therapist, to be attached to every mental hospital, which could cater to the need of a majority of such chronic patients who languished.

Schmidt[4] in his article, ‘A measurement of rehabilitation of psychiatric patients,’ comments that in psychiatry, as in general medicine, a full *restitutio ad integrum* cannot be expected even after the most efficient treatment, although, functioning can fortunately be restored after the disease. He has reported from a community-based mental health review in Sarawak, a state in the federation of Malaysia. They focused on patients in terms of rehabilitation of their previous working capacity. The rehabilitation status was measured by taking into consideration whether they were, ‘working’ ‘probably working,’ ‘Does some work,’ ‘Probably not working,’ and ‘Not working’. They assessed 584 consecutive patients visiting the clinic for follow-up and found that 82% of them were within the first three categories (‘Working’, ‘probably working’, ‘does some work’) and were functioning well, while 18% (‘Probably not working’ and ‘not working’) were not functioning, and therefore, could not claim to have been rehabilitated.

Nagaswamy *et al*,[5] carried out a very important assessment of the rehabilitation needs of schizophrenic patients. They interviewed 59 schizophrenic outpatients and their families to assess the subjective rehabilitative needs. They found that 64.4% wanted a job, 54.4% wanted some help for the family. Almost 90% of them desired rehabilitation in one form or another and most exhibited multiple needs, which emphasized the role of multifaceted, comprehensive, aftercare package programs. Even as the need for job as a priority was similar to findings in the west, this population differed by having low priority for social skills training and psycho-social structuring, in contrast to the west.

Channabasavanna,[2] in his editorial, has stated that early attempts at rehabilitation would have long-term benefits for the patients and their families. No treatment of mental disorder can be considered as complete or adequate without giving due consideration to rehabilitation or aftercare services. He has also commented that facilities, such as, pensions and other benefits and compensations that are forwarded to other major physical disorders and mental deficiency are not provided for major mental disorders.

Verma and Shiv[6] reported on the effect of rehabilitation in leprosy patients with psychiatric morbidity. They assessed 100 patients with leprosy, among whom 46 were rehabilitated and were staying in an ashram. Others were staying in a slum. They were assessed using the Goldberg General Health Questionnaire (GHQ) and Indian Psychiatric Interview Schedule (IPIS). They found statistically significant differences on psychiatric morbidity between the non-rehabilitated (85%) and rehabilitated groups (68%).

Gopinath and Rao[7] in their invited review article, have reviewed important world literature regarding psychiatric rehabilitation. They describe the principle, components, and efficacy of various rehabilitation activities. They have discussed the scenario in India and suggested steps to be taken to improve rehabilitation efforts in India.

Kastrup *et al*.,[8] have studied the psychological consequences of torture and they have described the principles of treatment. They have described a model for rehabilitation of such victims, being followed at their center at Copenhagen.

Mathai *et al*.,[9] in an unique, but small case control study, tried cognitive re-training of four detoxified male alcoholics and compared it with four controls. At the end of six weeks they found a significant improvement in information processing, memory, and reduction of neuro-psychological deficits. They concluded that neuropsychological rehabilitation was effective in improving cognitive defects of abstinent alcoholics.

Agarwal[10] in his review of the book, ‘Innovation in psychiatric rehabilitation,’ published by the Richmond Fellowship Society (India) comments, ‘Large rehabilitation facilities may be the only viable option’. He opines that there were many rehabilitative initiatives, but unfortunately most of them have not tried to evaluate their efforts scientifically as well as in economic terms.

Ponnuchamy *et al*.,[11] examined the role of family support groups in psychosocial rehabilitation. They observed that members attending support group meetings, expected to get more information about the illness, to develop skills to cope with problems at home, and to learn skills to deal with the ill person. They concluded that participation in a support group meeting positively affected key variables in the participant’s adaptation to mental illness in a relative.

Thara[12] in a commentary has stressed the need for cost-effectiveness studies for rehabilitation. She reports the experience of Schizophrenia Research Foundation (SCARF) in rural areas, where it was found that the most suitable elements of a rehabilitation program were empowering the families and offering simple, culture-specific interventions, such as distribution of livestock and fishing nets.

Kumar *et al*.,[13] have assessed the prevalence and pattern of mental disability among the rural population in Karnataka. It was a community-based, cross-sectional, house-to-house survey. They used Indian disability evaluation and assessment scale (IDEAS), developed by Rehabilitation committee of Indian psychiatric society (IPS). They studied one thousand subjects randomly. The prevalence of mental disability was found to be 2.3%. The prevalence was higher among females (3.1%) than among males (1.5%). The prevalence was the highest among the elderly and illiterates.

Suresh Kumar[14] observed that there is a definite limitation to the domains of social functioning, cognitive functioning, and psychopathology in chronic schizophrenia patients who have had no rehabilitation. Vocational rehabilitation significantly improves these limitations, which in turn helps these patients to integrate into the society so as to function efficiently in their roles.

## COMMENTS

Although rehabilitation of mentally ill is an essential component of psychiatric management there are very few publications in the Indian Journal of Psychiatry in this area. Although many NGOs are providing services in the area of rehabilitation, the models they follow and clinical benefits of the same are not published for the benefit of the psychiatric community. It is important that such rehabilitation service providers are encouraged to publish their activities. It is heartening that following the effort of the IPS to assess the disability in the mentally ill objectively, the government has extended disability benefits to psychiatric patients too. The reach of these benefits to the needy and their social consequences are yet to be assessed. Of late, there has been an initiative from the government of Karnataka to provide short-term residential care for recovering psychiatric patients. The effectiveness of such programs has to be further explored.

Also the heterogeneous population of India will have multiple unique needs and problems to be addressed. Although all mental health professionals stress the importance of culture-specific rehabilitative measures, the solution provided by them has not been published.

## CONCLUSION

There is a gross scarcity of publications in the IJP with respect to the rehabilitation in psychiatry. The mental health professionals need to take lead in publishing rehabilitation efforts or models being practiced by them in different parts of India. The effect of the disability benefits provided by the government needs to be assessed. The indigenous, innovative models followed need to be published.
